# Dietary guanidinoacetic acid supplementation favourably modulates energy dynamics and nitrogen balance in broilers

**DOI:** 10.1016/j.aninu.2026.05.001

**Published:** 2026-07-07

**Authors:** Nishchal K. Sharma, Sosthene Musigwa, Vivienne Inhuber, Alip Kumar, Stuart J. Wilkinson, David J. Cadogan, Shu-Biao Wu

**Affiliations:** a School of Environmental and Rural Science, University of New England, Armidale 2351, New South Wales, Australia; b Alzchem Trostberg GmbH, Dr.-Albert-Frank-Str. 83308, Trostberg, Germany; c Feedworks Pty Ltd., Romsey 3434, Victoria, Australia

**Keywords:** Apparent metabolizable energy, Broiler, Energy metabolism, Guanidinoacetic acid, Net energy, Nitrogen balance

## Abstract

The arginine-sparing potential of guanidinoacetic acid (GAA) has been well documented; however, information regarding its effects on energy dynamics and nitrogen balance in broilers is limited. This study evaluated the effects of dietary GAA supplementation on heat increment, net energy, efficiency of energy utilization, and nitrogen and energy balance in broilers using closed-circuit calorimetry chambers. One-d-old Ross 308 male broilers (39 ± 1 g, *n* = 80) were fed common starter and grower diets until 18 d of age. From d 19, birds continued to receive the grower diet supplemented with 0, 0.03%, 0.06%, or 0.09% GAA. Following an acclimation period in chambers (d 22–25), indirect calorimetry measurements were conducted from d 25 to 28. Each dietary treatment consisted of eight replicate chambers with 2 birds per chamber divided across two consecutive experimental runs. In each run, there were four replicate chambers per treatment. The efficacy of GAA on growth performance was evidenced by a linear improvement in feed conversion ratio (FCR; *P =* 0.022), along with a tendency for quadratic responses in weight gain (WG; *P =* 0.057) and feed intake (*P =* 0.080) as GAA levels increased. Dietary GAA did not affect (*P* > 0.05) apparent metabolizable energy (AME) or nitrogen-corrected AME (AMEn) of the feed. However, dietary net energy (NE) tended to increase linearly (*P =* 0.063) with increasing GAA inclusion. The efficiency of AME utilization for NE, expressed as NE/AME and NE/AMEn, was affected by GAA supplementation, with a linear increase in NE/AMEn (*P =* 0.017) and a tendency for a linear increase in NE/AME (*P =* 0.062). Dietary GAA linearly decreased (*P =* 0.031) AME intake per unit of WG while it had no impact (*P* > 0.05) on NE intake. Increasing dietary GAA levels increased nitrogen (N) retention in a quadratic manner (*P =* 0.017) and linearly improved N utilization efficiency (*P =* 0.002). Heat production decreased linearly (*P =* 0.015) with increasing GAA concentration. The retained energy as protein increased quadratically (*P =* 0.022) and retained energy as fat tended to decrease linearly (*P =* 0.090) with increasing GAA levels. In conclusion, dietary GAA reduced heat production and the efficiency of AME utilization for NE, and favourably modulated body energy partitioning by enhancing energy retention as protein, thereby improving feed efficiency in broilers.

## 1 Introduction

Energy and protein represent the two most costly components of broiler diets, and inefficiencies in their utilization not only limit growth performance but also increase feed costs and nitrogen excretion. Dietary energy alone accounts for approximately 60%–70% of total feed costs in broiler production. However, not all ingested energy is retained for growth, as a considerable proportion is dissipated as heat during the digestion, absorption and metabolism of nutrients in addition to excretion (Noblet et al., 2010). In broilers, it has been estimated that approximately 25% of metabolizable energy (ME) is lost as heat increment, although this proportion varies with nutrient type and feed ingredient composition (Carré et al., 2014; Wu et al., 2019). These energy losses reduce the efficiency with which dietary ME is converted into productive output, highlighting the importance of strategies to improve energy utilization efficiency. The net energy (NE) system accounts for energy losses associated with heat increment and is increasingly recognised as a more precise approach for evaluating dietary energy in poultry (Musigwa et al., 2021; Noblet et al., 2024; Wu et al., 2019). The ratio of NE to apparent metabolizable energy (AME) provides a measure of the efficiency with which AME is converted into NE (De Groote, 1974; Swick et al., 2013). Nutritional strategies that enhance the efficiency of energy and nitrogen utilization and reduce energy losses as heat are of increasing interest, as they offer the potential to conserve dietary energy and protein, improve feed efficiency and reduce feed costs in broiler production.

Creatine and its phosphorylated form, phosphocreatine, are readily available sources of energy in tissues with high and fluctuating energy demands, particularly in skeletal muscles (DeGroot et al., 2018). Growing broilers at high energy demand can utilize muscle phosphocreatine for rapid regeneration of adenosine triphosphate (ATP), thereby reducing reliance on oxidative metabolism of carbohydrates, lipids, and amino acids, processes that are associated with a higher heat increment (Wu et al., 2019). Guanidinoacetic acid (GAA) is a direct precursor of creatine and has been increasingly used as a feed additive to spare arginine for creatine synthesis in broiler (Barekatain et al., 2025; Dilger et al., 2013; Michiels et al., 2012; Sharma et al., 2022). Dietary GAA is absorbed into the bloodstream and methylated in the liver to form creatine, which is subsequently transported to tissues with high energy demands, such as skeletal muscles, where it is phosphorylated to phosphocreatine. Phosphocreatine serves as a rapidly mobilizable high-energy phosphate reservoir, replenishing ATP levels and maintaining muscle energy homeostasis (DeGroot et al., 2018). The GAA is also synthesized endogenously from arginine and glycine in the kidney; however, endogenously produced GAA and creatine can trigger a substantial metabolic drain on arginine availability. Supplementation with GAA can therefore reduce the endogenous requirement for arginine in creatine synthesis, allowing arginine to be redirected toward other essential metabolic functions such as muscle protein accretion, cell signalling, and hormone release rather than creatine formation (Portocarero and Braun, 2021) for which GAA is more efficient (DeGroot et al., 2018). Numerous studies have consistently reported improvements in feed efficiency and breast meat yield in broilers with GAA supplementation (Boney et al., 2020; Khajali et al., 2020; Sharma et al., 2022). Beyond its role in arginine sparing, GAA has been shown to exert direct effects on muscle energy metabolism, including increases in muscle creatine and phosphocreatine concentrations, improved phosphocreatine-to-ATP ratios, enhanced muscle glycogen storage, and improved glycogen-to-ATP ratios, all of which indicate an improved cellular energy status (DeGroot et al., 2019; Lemme et al., 2007; Michiels et al., 2012; Sharma et al., 2022). These metabolic and performance benefits have been observed in both arginine-deficient and arginine-adequate diets (DeGroot et al., 2019), suggesting that the growth promoting effects of GAA cannot be attributed solely to arginine replacement.

Some studies have demonstrated positive effects of GAA supplementation on broiler growth performance under reduced AME diets (Fosoul et al., 2018; Pirgozliev et al., 2022); however, improvements in AME following GAA supplementation have generally been inconsistent or non-significant. For example, Pirgozliev et al. (2022) reported no changes in measured AME corrected for nitrogen retention (AMEn) with GAA and Salgado et al. (2023) estimated an AME equivalency value of GAA using FCR rather than measuring the AME through the standard total excreta collection method. Despite growing evidence linking GAA supplementation to improved growth performance, the extent to which these responses are mediated through changes in whole-body energy metabolism and nitrogen utilization remains poorly understood. In particular, limited information is available on the actual energetic contribution of GAA and how GAA influences heat production, net energy, and the efficiency of energy and nitrogen utilization and their relationships with feed efficiency in broilers. Therefore, the present study aimed to investigate the effects of dietary GAA supplementation on net energy, energy utilization, nitrogen and energy balance in broilers using closed-circuit calorimetry chambers.

## 2 Materials and methods

### 2.1 Animal ethics statement

This study was approved by the Animal Ethics Committee of the University of New England (UNE), Australia (Approval No. ARA-2025–2699-4131). The experimental procedures were performed according to the guidelines for animal experiments set by the Australian Code for the Care and Use of Animals for Scientific Purposes (NHMRC, 2013).

### 2.2 Bird housing and management

For each of the two calorimetry runs, 40 male Ross 308 chicks (1-d-old, 39 ± 1 g) were sourced from Aviagen hatchery in Goulburn, New South Wales, Australia (total 80 birds were used in two runs). From d 0 to 21, birds were reared in floor pens in an environment-controlled poultry shed following Ross 308 management guidelines (Aviagen, 2022). At 22 d of age, two birds were transferred into each of 16 calorimetry chambers with lids open and air pumps running in a climate-controlled room for acclimatization. Following acclimatization, indirect calorimetry measurements were conducted from d 25 to 28. The acclimatization and measurement periods followed a standard timeframe previously used for net energy measurements (Swick et al., 2013; Wu et al., 2019). During this period, the amount of O_2_ consumption and CO_2_ production of broilers per chamber was determined to calculate heat production (HP) using the Brouwer equation (Brouwer, 1965). The respiratory quotient (RQ) was determined as the volume of CO_2_ produced divided by the volume of O_2_ consumed. The O_2_ consumption and CO_2_ production in each chamber were measured between 10:30 and 9:00 the next day for a total of three days. The calibration of the closed-circuit calorimetric chambers was performed using alcohol combustion a priori. Chambers that produced RQ values between 0.65 and 0.70 during calibration were considered acceptable for use in the experiments. The system was also checked daily for air leakage from the chambers before and during gas exchange measurements. If any leakage was detected, the respective chamber was reopened, the leak repaired, the chamber resealed, and the measurements resumed. Total excreta was collected daily and pooled for each chamber over 3 d. During this period, the excreta was stored in a cool room at 4 °C, and then excreta subsamples were stored in a freezer at −20 °C until further analysis. Daily body weight and daily feed intake per chamber were recorded, and FCR during the three-day experiment was calculated accordingly.

### 2.3 Diets and experimental design

The details of ingredients and nutrient composition of the experimental diets are presented in Table 1. The diets contained wheat, soybean meal, and canola meal as major ingredients and were formulated based on the Ross 308 nutrient specifications (Aviagen, 2022). The diets were thoroughly mixed and cold pelleted at 65 °C, with the starter diets crumbled. Birds received a common starter crumble for the first 10 d post–hatch, followed by a common 3 mm pelleted grower diet until d 18. The grower diet contained 12.77 MJ/kg AMEn, 21% crude protein (CP), 1.18% digestible lysine, and 1.07 digestible arginine to digestible lysine ratio. The diet contained phytase (Axtraphy Gold 10,000 TPT) at 1500 FTU/kg and xylanase (Axtra XB 201 TPT) from Dupont Animal Nutrition (Marlborough, UK). From d 19 to 28, birds continued to receive the grower diet supplemented with four different levels of GAA (Creamino, Alzchem Trostberg GmbH, Trostberg, Germany)—0, 0.03%, 0.06% or 0.09%. Each dietary treatment consisted of eight replicate chambers with 2 birds per chamber divided across two consecutive experimental runs. In each run, there were four replicate chambers per treatment. The two consecutive runs were conducted with a three-day gap to allow for the acclimatization period. The climate-controlled room housing the chambers was maintained at a uniform temperature and continuously monitored using temperature and humidity displays, with readings obtained from sensors installed inside each chamber. The average room temperature was 22 °C in both runs, and the variation between chambers ranged within ±1 °C (21–23 °C). Replicates were randomly distributed to ensure that any variation was evenly spread across all treatments.

**Table 1:** Ingredient and nutrient composition of the common starter and basal grower diets (%, as-is).

Items	Diets	
	Common starter	Basal grower
**Ingredients**		
Wheat	60.546	62.894
Soybean meal	27.800	25.500
Canola meal solvent	3.500	5.000
Meat and bone meal	3.000	0.000
Canola oil	1.900	3.200
L-Lysine HCl	0.410	0.333
DL-Methionine	0.396	0.342
L-Threonine	0.190	0.146
L-Valine	0.098	0.064
L-isoleucine	0.064	0.039
L-Arginine	0.100	0.079
Choline chloride (60%)	0.022	0.000
Vitamin premix^1^	0.080	0.080
Mineral premix^2^	0.100	0.100
Limestone	0.819	1.193
Salt	0.144	0.192
Sodium bicarbonate	0.335	0.253
Monocalcium phosphate	0.421	0.560
Phytase^3^	0.015	0.015
Xylanase^4^	0.010	0.010
Salinomycin	0.050	0.000
Total	100.000	100.000
**Nutrients**		
Dry matter	89.500	89.000
AMEn, MJ/kg	12.450	12.770
Crude protein	23.000	21.000
Dig lysine	1.320	1.180
Dig methionine	0.677	0.607
Dig methionine + cysteine	1.003	0.930
Dig threonine	0.884	0.795
Dig valine	1.003	0.910
Dig isoleucine	0.884	0.810
Dig arginine	1.400	1.263
Dig tryptophan	0.252	0.242
Calcium	0.960	0.840
Available phosphorus	0.500	0.420
Sodium	0.200	0.180
Choline, mg/kg	1700.000	1600.000

AMEn = apparent metabolizable energy corrected for nitrogen retention; Dig = digestible.

1 Vitamin premix per kg diet (UNE VM, Rabar Pty Ltd., Beaudesert, Queensland, Australia): vitamin A, 12 MIU; vitamin D, 5 MIU; vitamin E, 75 mg; vitamin K, 3 mg; nicotinic acid, 55 mg; pantothenic acid, 13 mg; folic acid, 2 mg; riboflavin, 8 mg; cyanocobalamin, 0.016 mg; biotin, 0.25 mg; pyridoxine, 5 mg; thiamine, 3 mg; antioxidant, 50 mg.

2 Mineral premix per kg diet (UNE TM, Rabar Pty Ltd., Beaudesert, Queensland, Australia): Cu, 16 mg as copper sulfate; Mn, 60 mg as manganese sulfate; Mn, 60 mg as manganous oxide; I, 0.125 mg as potassium iodide; Se, 0.3 mg; Fe, 40 mg, as iron sulfate; Zn, 50 mg as zinc oxide; Zn, 50 mg as zinc sulfate.

3 Axtraphy Gold 10,000 TPT (Dupont Animal Nutrition, Marlborough, UK).

4 Axtra XB 201 TPT (Dupont Animal Nutrition, Marlborough, UK).

### 2.4 Chemical analyses

Prior to diet formulation, the ingredients were analyzed for CP, ME, and amino acid contents using near-infrared spectroscopy (NIR systems, 6500, DK-3400, FOSS Analytical A/S, Hillerød, Denmark) standardized to Adisseo PNE Advanced calibration. Dry matter contents of GAA, diets and excreta samples were determined by placing the samples in a forced hot air oven (Thermoline, Thermo Electron Corp., Wetherill Park, Australia) at 105 °C following the method 934.01 (AOAC, 1990). For N analysis, frozen excreta samples were dried using a freeze-drier (Alpha 1-4 LDplus, Martin Christ Gefriertrocknungsanlagen GmbH, Osterode am Harz, Lower Saxony, Germany) and ground to pass through a 0.5-mm screen. Ground feed, excreta samples were analyzed in duplicate for N using the Dumas method (method 968.06) of AOAC (1990) with a LECO FP-2000 automatic N analyzer (Leco Corporation, St. Joseph, MI, USA). The N values on freeze-dried samples were converted on an oven (Thermoline) dry matter (DM) basis. The analyzed N values were corrected for CP by multiplying the correction factor of 6.25. The gross energy in GAA, feed, and excreta was measured using a 0.5 g sample in an adiabatic bomb calorimeter (IKA Werke, C7000, GMBH and Co., Staufen, Germany) with benzoic acid as the standard. Around 500 g of feed was collected from each treatment and shipped to Alzchem Trostberg GmbH lab in Germany to measure GAA concentrations. The CO_2_ trapped in the KOH solution was recovered using the barium chloride (BaCl_2_) precipitation technique as described previously (Swick et al., 2013).

### 2.5 Calculation

The calculations are based on the papers published previously on net energy measurements (Wu et al., 2019; Noblet et al., 2022). In short,

The nitrogen balance data are expressed as g/bird per d and calculated as:

$$RN=N_{i}\;–\;N_{e}\text{,}$$

where RN is the retained nitrogen in the body; *N*_*i*_ is the nitrogen intake from the diet (g/bird per d) and *N*_*e*_ is the nitrogen excreted (g/bird per d).

$$\text{Nitrogen}\;\text{utilization}\;\text{efficiency}=RN/N_{i}\times 100\text{.}$$

The AME values of diets were determined by the total collection method previously described by Bourdillon et al. (1990) with modifications. The equation used was:

AME(MJ/kgDM)=[(FeedGE×FI)–(ExcretaGE×Totalexcretaoutput)]/FI,

where GE is the gross energy (MJ/kg); FI is the feed intake (g/bird per d DM).

The AMEn values of the diets were determined using the following equations:

AMEn(MJ/kgDM)=AME–[8.22×RN]/FI,

where 8.22 kcal/g is the nitrogen correction factor for each gram of nitrogen retained in the body corresponding to the energy content of uric acid per g of N (Hill and Anderson, 1958).

DailyAMEintake(kJ/birdperd)=AME×FI(g/d).

Heat production values were estimated from the volumes of CO_2_ expired and O_2_ consumed by birds in the chamber using the modified Brouwer (1965) equation (excluding methane and N in expired gas from the equation due to the negligible amount of these produced in avian species) as follows:

$$\text{HP}\;\left(\text{kJ}\right)=16.18\times {\text{VO}}_{2}\;\left(L\right)+5.02\times {\text{VCO}}_{2}\;\left(L\right)\text{,}$$

where VO_2_ and VCO_2_ are volumes of O_2_ consumed and CO_2_ expired, respectively.

The RQ of the birds was calculated as the ratio of the CO_2_ volume exhaled to the O_2_ volume consumed by birds.

Heat increment (HI) of feed was calculated as:

HI(kJ/kg)=(HP–FHP)/FI(kg),

where FHP is the fasting HP. The FHP value of 450 kJ/kg per metabolic body weight (BW^0.70^) per d for broilers reported by Noblet et al. (2015) was used in the calculation. This FHP value corresponds to the asymptotic HP (at zero activity) during a 24-h fasting period. The NE values of feeds were calculated using the following equations:

NE(MJ/kgDM)=(AMEintake–HI)/FI.

Retained energy (RE) was calculated as:

REtotal(kJ)=AMEintake–HP,

REasprotein(kJ/gperd)=RN(g/d)×6.25×23.8(kJ/gprotein),

where RN is the retained N, 6.25 is the protein equivalent of 1 g N, and 23.8 is the energy equivalent of 1 g body protein;

REasfat(kJ/gperd)=RE–REasprotein.

The results for the AME intake, NE intake, HP, RE, RE as protein and RE as fat are expressed as kJ/kg BW^0.70^ per d. Feed conversion ratio (FCR) was calculated as feed intake (g DM/bird per d) divided by weight gain (WG, g/bird per d).

### 2.6 Statistical analysis

Data were analyzed as one-way ANOVA using JMP statistical software v14 (SAS Institute Inc, Cary, NC). Orthogonal polynomial contrasts, with run included as a covariate, were used to test linear and quadratic effects of increasing levels of GAA using the JMP software. “Run” was included in the statistical model as a covariate to account for potential variation between the two experimental periods that was unrelated to dietary treatment. Although environmental conditions, chamber settings, bird management, and experimental procedures were maintained as consistently as possible between runs, minor temporal differences (e.g., room conditions, bird variation, or day-to-day operational variation) may still occur in calorimetry studies. Including run in the model therefore helps partition this non-treatment variation and improves the precision of treatment effect estimation. Treatments were randomized within each run and equally distributed across runs, minimizing confounding between treatment and run effects. The statistical model used was as follows:

$$Y_{ij}=\beta_{0}+\beta_{1}X_{ij}+\beta_{2}X_{ij}^{2}+\beta_{3}Run_{j}+\varepsilon_{ij}\text{,}$$

where *Y*_*ij*_ is the response variable; *β*_*0*_ is the intercept; *X*_*ij*_ is the GAA level; *X*^*2*^_*ij*_ is the quadratic (second-order) term for GAA; *β*_*1*_ is the linear effect of GAA; *β*_*2*_ is the quadratic effect of GAA; *Run*_*j*_ is the covariate term for run; *β*_*3*_ is the effect of run; *ε*_*ij*_ is the residual error term.

The calorimetry chamber served as the experimental unit (*n* = 32). Partial correlation analysis, with run included as a covariate, was used to determine the relationships between nitrogen and energy balance parameters and FCR. Statistical significance is declared at *P* < 0.05, and trends toward significance are considered when 0.05 < *P* < 0.10.

## 3 Results

The analyzed nutrient composition and GAA concentrations of the experimental grower diets are presented in Table 2. The calculated and analyzed CP levels in the diets were similar (about 21%). The analyzed GAA contents in the diets closely matched the calculated inclusion levels suggesting that Creamino provided the desired levels of GAA in the diets, considering a minimum of 96% GAA concentration in Creamino.

**Table 2:** Analyzed composition of experimental grower diets and Creamino (as-is).

Items	Creamino^1^	Diets^2^			
		GAA-0	GAA-0.03%	GAA-0.06%	GAA-0.09%
Dry matter, %	99.8	86.8	87.2	87.4	87.5
Crude protein, %		20.6	21.4	21.3	21.5
Non-protein nitrogen, %	34.05				
Gross energy, MJ/kg	13.96	16.63	16.73	16.72	16.73
GAA concentration, %	96.0000	<RL^3^	0.0238	0.0539	0.0869

GAA = guanidinoacetic acid; RL = reporting limit.

1 Creamino is a trade name for GAA manufactured by Alzchem Trostberg GmbH, Trostberg, Germany.

2 A common grower diet was formulated and GAA was added at 0, 0.03%, 0.06% and 0.09% over the top.

3 Reporting limit set to 0.002% for GAA.

### 3.1 Growth performance

The effect of GAA supplementation on growth performance of broilers from 25 to 28 d is presented in Table 3. On average, the body weight of the birds at the start of the experiment (d 25) was 1541 g, which was approximately 10% higher than the expected body weight for Ross 308 males at the same age. There were no differences (*P* = 0.794) in mean body weight among treatments at the start of the experiment.

**Table 3:** Effects of guanidinoacetic acid (GAA) supplementation on dietary energy, energy utilization efficiency, and growth performance of broilers (d 25-28).

Items	Diets^1^				SEM	*P*-value		
	GAA-0	GAA-0.03%	GAA-0.06%	GAA-0.09%		ANOVA	Linear	Quadratic
**Growth performance^2^**								
Mean BW at d 25, g/bird	1537	1533	1558	1536	9.8	0.794	0.807	0.629
FI, g/bird per d (DM)	125.6	126.7	128.1	122.6	1.12	0.166	0.334	0.080
WG, g/bird per d	106.4	112.2	113.0	109.5	1.61	0.142	0.326	0.057
FCR, g/g (DM)	1.184	1.133	1.137	1.121	0.0113	0.038	0.022	0.323
AME intake, kJ/bird per d	1813	1830	1852	1774	13.7	0.144	0.346	0.055
NE intake, kJ/bird per d	1362	1356	1409	1338	12.7	0.125	0.832	0.119
AME intake/WG, kJ/g	17.10	16.36	16.43	16.24	0.173	0.057	0.031	0.294
NE intake/WG, kJ/g	12.84	12.11	12.51	12.24	0.121	0.060	0.122	0.268
**Energy, MJ/kg DM^3^**								
AME	14.44	14.44	14.45	14.48	0.031	0.881	0.612	0.795
AMEn	13.55	13.52	13.53	13.54	0.032	0.961	0.909	0.695
NE	10.84	10.70	10.99	10.92	0.041	0.021	0.063	0.569
Heat increment of feed	3.60	3.75	3.45	3.56	0.042	0.013	0.114	0.718
**Energy utilization efficiency^4^**								
AME/GE	0.754	0.753	0.755	0.758	0.0021	0.585	0.389	0.527
AMEn/GE	0.708	0.705	0.707	0.709	0.0022	0.763	0.813	0.450
NE/AME	0.751	0.741	0.761	0.754	0.0023	0.006	0.062	0.624
NE/AMEn	0.800	0.791	0.812	0.806	0.0031	0.004	0.017	0.711

DM = dry matter; BW = body weight; FI = feed intake; WG = weight gain; FCR = feed conversion ratio; AME = apparent metabolizable energy; AMEn = AME corrected for nitrogen retention; NE = net energy; GE = gross energy; SEM = standard error of the mean.

1 A common grower diet was formulated and GAA was added at 0, 0.03%, 0.06% and 0.09% over the top.

**Fig. 1:**
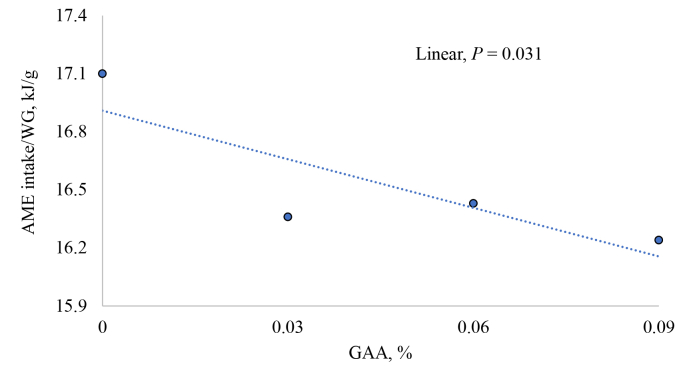
Effects of guanidinoacetic acid (GAA) supplementation on apparent metabolizable energy (AME) intake per unit of weight gain (WG) of broilers (d 25-28).

Increasing dietary GAA levels resulted in a linear improvement in FCR (*P =* 0.022) by a magnitude of up to 6 points. The diet with no GAA had the worst FCR (1.184) which gradually improved to an FCR of 1.121 at 0.09% GAA inclusion level. There was a tendency toward quadratic responses in daily WG (*P =* 0.057) and feed intake (*P =* 0.080) to the GAA dosage, both of which increased up to a level of 0.06% GAA and decreased thereafter. Dietary GAA supplementation tended to affect daily AME intake per bird (quadratic, *P =* 0.055) and linearly decreased (*P =* 0.031) AME intake per unit of WG (Fig. 1). The AME intake per unit of WG was highest from the diet with no GAA (17.10 kJ/g) and gradually decreased to 16.24 kJ/g with 0.09% GAA. However, there was no effect of GAA (*P* > 0.05) on daily NE intake per bird and NE intake per unit of WG.

### 3.2 Energy value of feed and energy utilization efficiency

The effect of GAA supplementation on the energy value of feed and energy utilization efficiency in broilers is presented in Table 3. Dietary GAA did not affect (*P* > 0.05) AME or AMEn of the feed. However, dietary NE tended to increase linearly (*P =* 0.063). The efficiency of AME utilization for NE, expressed as NE/AME and NE/AMEn, was affected by GAA supplementation, with a linear increase in NE/AMEn (*P =* 0.017) and a tendency for a linear increase in NE/AME (*P =* 0.062). The NE/AMEn linearly increased from 0.800 at 0 GAA to 0.806 at 0.09% GAA. The efficiency of GE utilization for AME, expressed as AME/GE and AMEn/GE, was not affected (*P* > 0.05) by GAA supplementation.

### 3.3 Nitrogen and energy balance

**Fig. 2:**
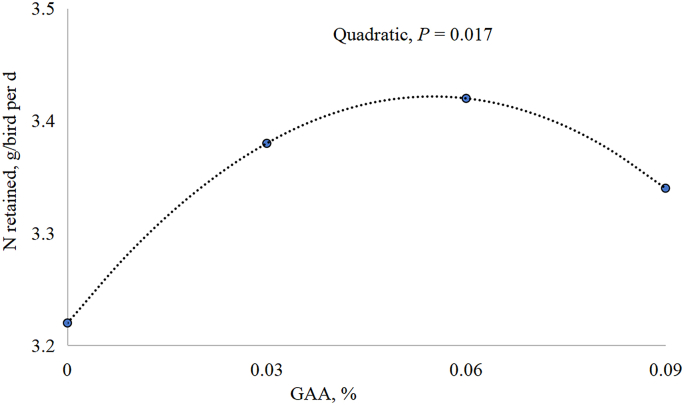
Effects of guanidinoacetic acid (GAA) supplementation on nitrogen (N) retention in broilers (d 25-28).

**Fig. 3:**
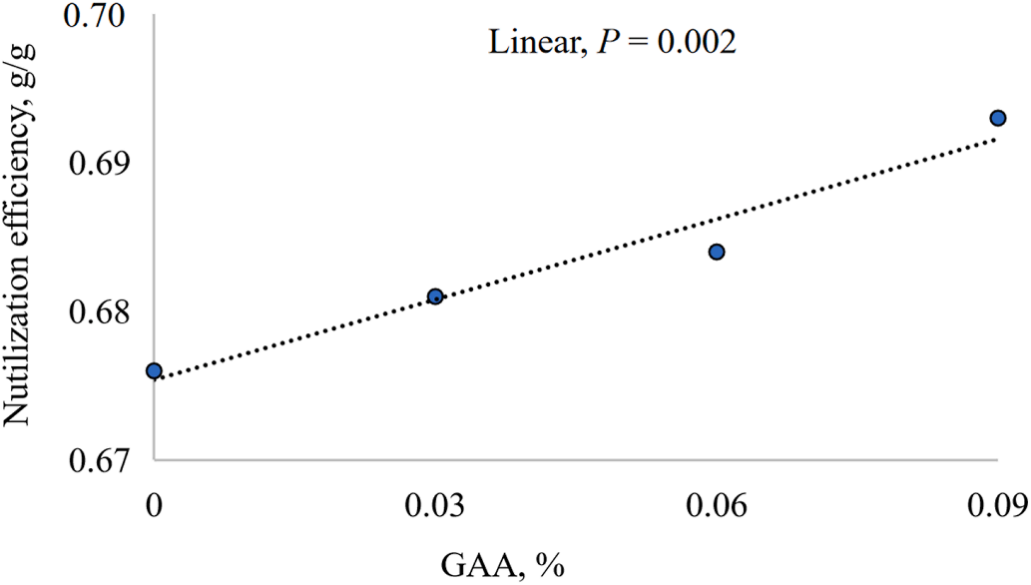
Effects of guanidinoacetic acid (GAA) supplementation on nitrogen (N) utilization efficiency in broilers (d 25–28).

**Fig. 4:**
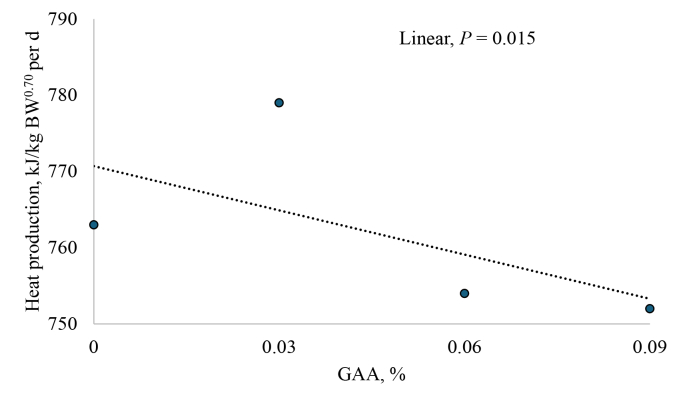
Effects of guanidinoacetic acid (GAA) supplementation on heat production in broilers (d 25–28).

**Fig. 5:**
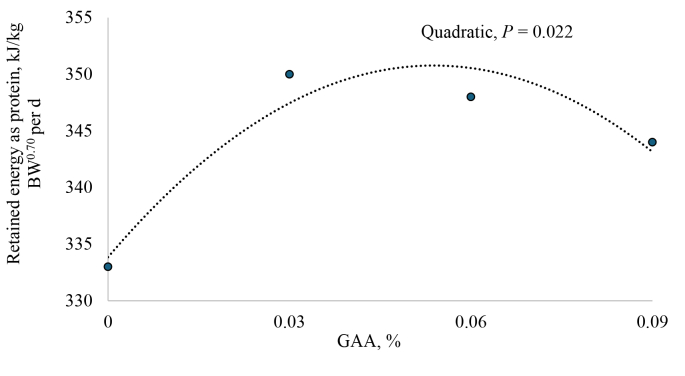
Effects of guanidinoacetic acid (GAA) supplementation on retained energy as protein in broilers (d 25–28).

The nitrogen and energy balances of birds were affected by dietary GAA supplementation (Table 4). Increasing dietary GAA levels increased N retention in a quadratic manner (*P =* 0.017; Fig. 2) with a retention of 3.22 g/bird/d at 0 GAA to 3.42 and 3.34 g/bird/d at 0.06% and 0.09% GAA respectively. There was a linear improvement in N utilization efficiency with increasing levels of GAA (*P =* 0.002; Fig. 3) from 0.676 at 0 GAA to 0.693 at 0.09% GAA. Heat production decreased linearly (*P =* 0.015) with increasing GAA concentration (Fig. 4) from 763 kJ/kg BW^0.70^ at 0 GAA to 752 kJ/kg BW^0.70^ at 0.09% GAA. The AME intake tended to change quadratically (*P =* 0.066), but there was no effect on NE intake (*P* > 0.05) with increasing levels of GAA. There was no effect (*P* > 0.05) of GAA on total RE but the RE as protein increased quadratically (*P =* 0.022; Fig. 5) while RE as fat tended to decrease linearly (*P =* 0.090) with increasing GAA levels. There was no effect of GAA (*P* > 0.05) on RQ. The correlation between nitrogen and energy balance parameters and FCR is presented in Table 5. The nitrogen utilization efficiency in broilers was negatively correlated with FCR (*r* = –0.469, *P =* 0.008). Similarly, retained energy as protein was negatively correlated with FCR (*r* = –0.420, *P =* 0.019) and retained energy as fat had no relationship with FCR (*P* > 0.05).

**Table 4:** Effects of guanidinoacetic acid (GAA) supplementation on nitrogen and energy balance of broilers (d 25-28).

Items	Diets^1^				SEM	*P*-value		
	GAA-0	GAA-0.03%	GAA-0.06%	GAA-0.09%		ANOVA	Linear	Quadratic
**Nitrogen balance, g/bird per d**								
Intake	4.77	4.97	4.99	4.82	0.042	0.089	0.553	0.014
Retained	3.22	3.38	3.42	3.34	0.033	0.033	0.063	0.017
Nitrogen utilization efficiency	0.676	0.681	0.684	0.693	0.0022	0.012	0.002	0.543
**Energy balance, kJ/kg BW^0.70^ per d**								
HP	763	779	754	752	3.3	0.005	0.015	0.115
AME intake	1259	1271	1269	1230	7.1	0.123	0.139	0.066
NE intake	945	941	966	927	6.5	0.160	0.550	0.141
RE total	495	491	516	477	6.5	0.159	0.547	0.140
RE as protein	333	350	348	344	2.3	0.033	0.075	0.022
RE as fat	162	142	168	133	5.4	0.019	0.090	0.407
**RQ**	1.058	1.045	1.050	1.041	0.0032	0.315	0.132	0.779

AME = apparent metabolizable energy; NE = net energy; RE = retained energy; BW = body weight; BW^0.70^ = metabolic body weight; HP = heat production; RQ = respiratory quotient; SEM = standard error of the mean.

1 A common grower diet was formulated and GAA was added at 0, 0.03%, 0.06% and 0.09% over the top.

**Table 5:** Correlation between nitrogen and energy balance parameters and feed conversion ratio (FCR).

Parameters	FCR, g/g (DM)
Nitrogen utilization efficiency	*r* = −0.469, *P =* 0.008
RE as protein, kJ/kg BW^0.70^ per d	*r* = −0.420, *P =* 0.019
RE as fat, kJ/kg BW^0.70^ per d	*r* = 0.163, *P =* 0.380

DM = dry matter; RE = retained energy; BW^0.70^ = metabolic body weight.

## 4 Discussion

Guanidinoacetic acid has been predominantly used in broiler feed as an alternative or partial replacement for arginine, with reported arginine equivalency values ranging from 75% to 150%, depending on the response criterion evaluated (DeGroot et al., 2018; Dilger et al., 2013; Michiels et al., 2012; Sharma et al., 2022). While the arginine-sparing effects of GAA are well established, its contribution to whole-body energy and nitrogen balance and the extent to which it may exert an “energy-sparing” effect in broilers remains poorly understood.

The analyzed GE content of GAA used in the present study was 3336.4 kcal/kg (13.96 MJ/kg). At practical inclusion levels of 0.06%–0.09%, GAA contributes only approximately 2 to 3 kcal/kg of GE to the diet, which is nutritionally negligible. In the present study, GAA supplementation did not influence the AME and AMEn values of the diets and therefore an AME equivalency for GAA could not be established. Several studies have suggested that GAA may improve energy utilization in broilers; however, the AME equivalency of GAA has not been measured precisely using standard methods, and in most cases improvements in AME with GAA supplementation were not significant, similar to the findings of the present study. Fosoul et al. (2018) and Pirgozliev et al. (2022) reported improvements in growth performance when GAA was added to diets with reduced ME content. Notably, Pirgozliev et al. (2022) demonstrated that supplementation with 0.06% GAA restored the growth performance of broilers fed diets reduced by 50 to 100 kcal/kg AME although AMEn was not affected by GAA supplementation. Salgado et al. (2023) estimated an AME equivalency of 88.5 kcal/kg for 0.06% GAA using the equivalence equation for FCR rather than measuring the AME using the standard total excreta collection method. Despite the absence of changes in dietary AME and AMEn in the present study, GAA significantly improved the efficiency of AME utilization for NE (NE/AME and NE/AMEn). This was due to the high net energy value of feed with GAA supplementation, which is directly related to lower HP. Dietary GAA supplementation also linearly reduced AME intake per unit of WG. Collectively, these findings indicate that the benefits of GAA may be realised not through increasing the AME value of the diet per se, but through enhancing the efficiency with which dietary AME is utilized for NE and growth, thereby contributing to reduced feed costs in broiler production.

The improvement in energy utilization efficiency observed in this study was accompanied by a reduction in HP with increasing levels of GAA. As the immediate precursor of creatine, GAA supplementation is expected to increase tissue creatine, phosphocreatine and ATP availability (DeGroot et al., 2019; Lemme et al., 2007). Improved phosphocreatine availability allows rapid regeneration of ATP with a lower reliance on oxidative metabolism of carbohydrates, lipids and amino acids, processes that are associated with a high heat increment (Wu et al., 2019). Consequently, the energetic cost of ATP resynthesis may be reduced, resulting in lower endogenous HP as observed in the present study. These mechanisms could collectively improve the efficiency with which AME is utilized as NE for growth, particularly in energetically demanding tissues such as skeletal muscle. Enhanced energy efficiency was also reflected in altered body energy partitioning. Increasing dietary GAA levels resulted in greater energy retention as protein and reduced energy retention as fat. Interestingly, RQ was not affected by GAA supplementation despite its effect on energy partition in the birds. The shift of energy partitioning towards protein indicates that GAA improved the efficiency of amino acid utilisation, reducing their catabolism for energy and favouring their incorporation into body protein, which implies increased RQ potential. However, the reduced fat retention indicates more fat was catabolized implying reduced RQ. Therefore, GAA supplementation resulted in unchanged RQ level. The improved protein accretion likely contributed to the observed increases in nitrogen retention and nitrogen utilization efficiency in the present study. These findings are supported by previous works which demonstrated increased breast meat yield and reduced abdominal fat pad weight in broilers supplemented with GAA (Boney et al., 2020; Sharma et al., 2022). Collectively, these responses suggest that GAA promotes a shift in nutrient partitioning towards lean tissue deposition. This shift may have been driven not only by an improved cellular energy status but also by the arginine-sparing effect of GAA, which bypasses the arginine and glycine-dependent step of endogenous GAA synthesis. By conserving arginine and glycine for muscle protein accretion and reducing amino acid catabolism to meet ATP demands during rapid muscle growth, GAA supplementation may have decreased nitrogen losses associated with deamination and uric acid synthesis. Consequently, a greater proportion of dietary nitrogen is retained in productive tissue leading to improved nitrogen utilization efficiency as observed in the present study. Furthermore, given the small proportion of GAA supplementation in the feed, energy and nitrogen provided by GAA per se are limited. Therefore, the improvements in energy and nitrogen observed in the current study may be mediated by a sequential cascade of cellular metabolic reactions, which warrants further study.

Consistent with these metabolic responses, a linear improvement in FCR was observed as dietary GAA increased from 0.03% to 0.09%, with improvements ranging from 4.7 to 6.3 points. This response corroborates previous reports identifying FCR as the most consistently improved performance parameter in broilers supplemented with GAA (Khajali et al., 2020; Sharma et al., 2022). The positive correlations between feed efficiency, nitrogen utilization efficiency, and energy retained as protein observed in the present study further support the premise that GAA enhances growth performance primarily by improving the efficiency of energy and nitrogen utilization. Collectively, these findings suggest that GAA facilitates a more favourable partitioning of dietary nutrients towards lean tissue deposition, reducing energy losses as heat and nitrogen losses via excretion, and thereby providing a mechanistic basis for the robust improvements in feed efficiency associated with GAA supplementation. While the findings of this study are directly applicable to male birds, the underlying physiological responses are expected to be relevant in mixed sex flock. However, it is acknowledged that sex-specific responses may occur and warrant investigation in future studies.

## 5 Conclusion

This study demonstrated that dietary supplementation with GAA favourably modulates whole-body energy dynamics and nitrogen balance in broilers. GAA improved the efficiency with which dietary energy and nitrogen were utilized for growth. These improvements were mediated through reduced HP, enhanced AME efficiency for NE, and a shift in energy partitioning towards protein accretion at the expense of fat deposition. The cumulative effect of these responses was a clear, dose-dependent improvement in feed efficiency. Collectively, these findings indicate that the benefits of GAA extend beyond its arginine-sparing role, highlighting its function in reducing HP and enhancing both energy and nitrogen utilization efficiency, thereby promoting lean tissue accretion. Further study may be required to elucidate the mechanisms by which GAA improves the energy and nitrogen efficiencies to the extent shown in the current and previous studies. It is speculated that GAA may trigger a sequential cascade of metabolic reactions in cells leading to magnified efficiency of energy and nitrogen utilization.

## Credit Author Statement

**Nishchal K. Sharma:** Writing – original draft, Visualization, Resources, Methodology, Investigation, Funding acquisition, Formal analysis, Data curation, Conceptualization. **Sosthene Musigwa:** Writing – review & editing, Methodology, Investigation. **Vivienne Inhuber:** Writing – review & editing, Methodology. **Alip Kumar:** Writing – review & editing, Investigation. **Stuart J. Wilkinson:** Writing – review & editing. **David J. Cadogan:** Writing – review & editing, Methodology. **Shu-Biao Wu:** Writing – review & editing, Visualization, Validation, Supervision, Methodology, Investigation, Data curation.

## Declaration of competing interest

The authors declare the following financial interests/personal relationships which may be considered as potential competing interests: Dr. Vivienne Inhuber is currently employed by Alzchem Trostbertg GmbH, Trostberg, Germany; Stuart J. Wilkinson and David J. Cadogan are currently employed by Feedworks Pty Ltd., Romsey, Victoria, Australia; the research project is funded by Alzchem Trostbert GmbH, Trostbertg, Germany. Shu-Biao Wu is an Editorial Board Member for Animal Nutrition and was not involved in the editorial review or the decision to publish this article.
